# DMSO-tolerant ornithine decarboxylase (ODC) tandem assay optimised for high-throughput screening

**DOI:** 10.1080/14756366.2022.2150186

**Published:** 2022-11-30

**Authors:** Mingu Gordon Park, Suyeon Yellena Kim, C. Justin Lee

**Affiliations:** a KU-KIST Graduate School of Converging Science and Technology, Korea University, Seoul, South Korea; b Center for Cognition and Sociality, Institute for Basic Science (IBS), Daejeon, South Korea

**Keywords:** Ornithine decarboxylase, Dimethyl sulfoxide, cucurbit[6]uril, trans-4-(4-(dimethylamino)-styryl)-1-methylpyridinium iodide, high-throughput screening assay

## Abstract

Ornithine decarboxylase (ODC), the first rate-limiting enzyme in polyamine synthesis, has emerged as a therapeutic target for cancer and Alzheimer’s disease (AD). To inhibit ODC, α-difluoromethylornithine (DFMO), an irreversible ODC inhibitor, has been widely used. However, due to its poor pharmacokinetics, the need for discovery of better ODC inhibitors is inevitable. For high-throughput screening (HTS) of ODC inhibitors, an ODC enzyme assay using supramolecular tandem assay has been introduced. Nevertheless, there has been no study utilising the ODC tandem assay for HTS, possibly due to its intolerability to dimethyl sulfoxide (DMSO), a common amphipathic solvent used for drug libraries. Here we report a DMSO-tolerant ODC tandem assay in which DMSO-dependent fluorescence quenching becomes negligible by separating enzyme reaction and putrescine detection. Furthermore, we optimised human cell-line-based mass production of ODC for HTS. Our newly developed assay can be a crucial first step in discovering more effective ODC modulators than DFMO.

## Introduction

Ornithine decarboxylase (ODC, EC 4.1.1.17) is a pyridoxal-5′-phosphate-dependent enzyme responsible for conversion of L-ornithine into putrescine and carbon dioxide^1–3^. Elevated levels of ODC and polyamines have long been associated with cancer and other hyperproliferative diseases^4^^,^^5^. Notably, our group has recently shown that ODC inhibition turns toxic severe reactive astrocytes into beta-amyloid (Aβ)-detoxifying astrocytes in Alzheimer’s disease (AD) model and raised ODC inhibition as a promising therapeutic strategy for AD^6^. Inhibiting ODC has been proposed to interfere with polyamine biosynthesis, because ODC is required for the first step in polyamine biosynthesis.^3^^,^^4^^,^^7–9^. The most widely used irreversible ODC inhibitor is α-difluoromethylornithine (DFMO) which has been developed and FDA-approved for the treatment of West African sleeping sickness (trypanosomiasis)^10–13^ and hirsutism^14^^,^^15^. In addition, there are ongoing clinical trials evaluating the use of DFMO in cancer including neuroblastoma as well as cancer-unrelated human diseases, such as AD, diabetes and Bachmann–Bupp syndrome (OMIM: 619075)^16–19^. However, the *in vivo* efficacy of DFMO is decreased by its poor pharmacokinetics due to fast excretion and the relatively short half-life of ODC^7^^,^^20^. Moreover, the covalent bonding between DFMO and ODC unexpectedly increases the binding affinity between ODC and ODC antizyme 1 (OAZ1), which accelerates the clearance of the irreversibly inhibited ODC and upregulates the compensatory production of fresh ODC^21–23^. Therefore, it is essential to develop better human ODC inhibitors than DFMO.

**Figure 1.**
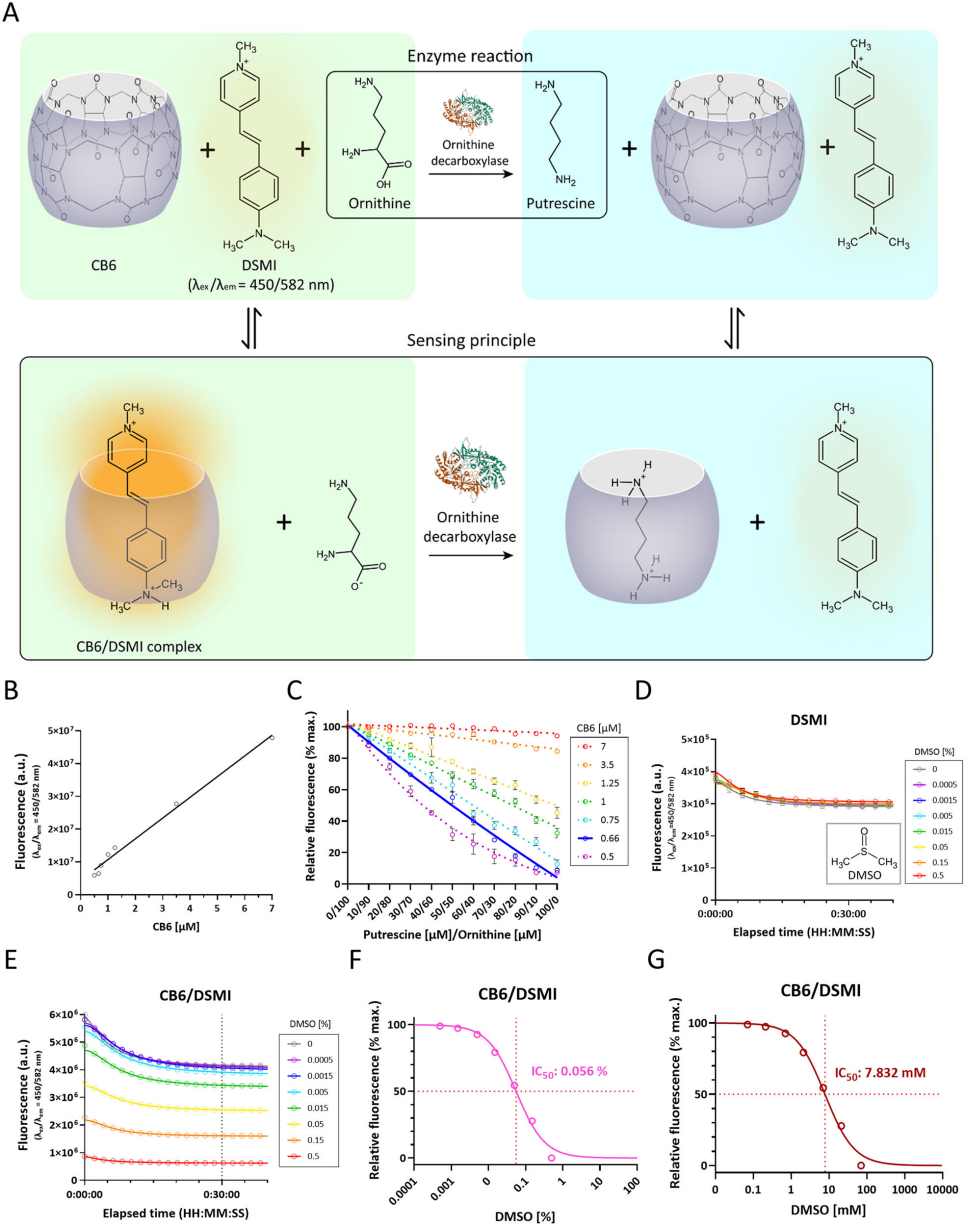
CB6/DSMI complex is sensitive to DMSO at high concentrations. A. Working principle of the ODC tandem assay. B. A linear relationship between the CB6 concentration and the fluorescence of CB6/DSMI complex. C. Standard calibrations with putrescine and ornithine at varying concentrations to determine the optimal CB6 concentration for 4 µM DSMI. D. Time-course plots of the fluorescence of DSMI with DMSO at different concentrations. E. Time-course plots of the fluorescence of CB6/DSMI with DMSO at different concentrations. F. A dose-response curve of the fluorescence quenching of CB6/DSMI by DMSO [%]. G. A dose-response curve of the fluorescence quenching of CB6/DSMI by DMSO [mM].

Based on these desperate needs, there have been numerous efforts to explore novel ODC inhibitors^23–25^. Furthermore, to meet the need for high-throughput screening (HTS)-compatible *in vitro* ODC assays, a label-free, continuous and fluorescence-based ODC assay has been introduced for HTS of ODC inhibitors^26^^,^^27^. This method has been referred to as tandem assay which exploits a differential, reversible, and competitive intermolecular binding of three potential guests (substrate, product, and dye) to a synthetic receptor^28^^,^^29^. The ODC tandem assay requires reporter pairs composed of cucurbit[6]uril (CB6) as a supramolecular macrocyclic receptor and trans-4-[4-(dimethylamino)styryl]-1-methylpyridinium iodide (DSMI) as a fluorescent dye. DSMI binds to the macrocyclic cavity of CB6 to form a highly fluorescent inclusion complex (Figure 1A). Because putrescine competes strongly with DSMI for the macrocyclic cavity of CB6, putrescine production via the L-ornithine decarboxylation continuously displaces DSMI with time and results in a concomitant decrease in fluorescence intensity^27^ (Figure 1A). This tandem assay appears to be the best way for adaptation to HTS, because it has been claimed to be simple, inexpensive, rapid, sensitive. However, there has been no study utilising the ODC tandem assay for HTS of compound libraries dissolved in dimethyl sulfoxide (DMSO), a common amphipathic solvent widely used for the addition of compounds to be tested in drug discovery, possibly due to binding of DMSO to CB6^30^. Therefore, it is highly necessary to develop a reliable ODC tandem assay by minimising the DMSO-dependent fluorescence quenching of the CB6/DSMI reporter pairs for efficient discovery of novel ODC inhibitors in a high-throughput manner.

HTS requires a large amount of human ODC enzymes. To obtain human ODC on a large scale, several studies performed overexpression of recombinant human *ODC1* in *Escherichia coli* (*E. coli*)^31^^,^^32^. However, it has been generally accepted that *E. coli*-based recombinant protein production does not provide appropriate post-translational modifications (PTMs) for mammalian proteins^33–36^. Purifying native human ODC from the human brain or peripheral tissues can be another option. There was even a study that performed ODC enzyme assay with endogenous human ODC extracted from human foetal liver^37^. However, it is time-consuming and labour-intensive for the extraction of native ODC from human tissue. To overcome these limitations, mammalian cell-based mass production of recombinant human ODC with proper folding and PTMs, which has never been attempted before, is necessary for application to HTS of human ODC inhibitors.

In this study, to establish a DMSO-tolerant ODC tandem assay in the microplate format for HTS applications, we firstly determined a DMSO concentration threshold below which DMSO-dependent fluorescence quenching of the CB6/DSMI complexes was negligible. To develop a method for mass production of human ODC, we utilised the Expi293F^TM^ expression system, Twin-Strep-tag/Strep-Tactin chromatography and size-exclusion chromatography. Using the purified human ODC, we established an optimised protocol which minimises DMSO-dependent fluorescence quenching of the CB6/DSMI complexes. This optimised protocol enables us to keep using DMSO as a universal solvent to dissolve drug candidates, thereby allowing exploration of large libraries of potential ODC inhibitors.

## Materials and methods

### Overexpression of recombinant human ODC in Expi293F^TM^ cells

Human ODC1 transcript variant 1 (Origene, RG206858, 1383 bp) was amplified by PCR using Q5^®^ High-Fidelity DNA Polymerase (NEB, M0491S) and the flowing primers: EcoRI_ODC1 (forward) 5′-CCGGAATTCATGAACAACTTTGGTAATGAAG-3′; AGEI_ODC1 (reverse) 5′-ATTTACCGGTCACATTAATACTAGCCGAAGCAC-3′. The PCR reaction mix was optimised according to manufacturer’s protocol. After initial denaturation (98 °C for 1 min), 32 cycles (98 °C for 10 s, 69 °C for 30 s, and 72 °C for 30 s) were performed, followed by a final extension (72 °C for 2 min). The resulting PCR product was cloned into EcoRI/AgeI-cut pHR-CMV-TetO2_3C-Twin-Strep_IRES-EmGFP (Addgene, 113884) by using T4 ligase (Enzynomics, M001H). The recombinant human ODC was overexpressed using Expi293F^TM^ expression system (Thermo Fisher Scientific, A14635). Just prior to transient transfection, 5.4 × 10^8^ cells of Expi293F^TM^ were added to 180 mL of Expi293^TM^ Expression Medium (3 × 10^6^ cells/mL) in a 1-L flask with vent cap (Corning, CC-431147) incubated at 125 rpm, 8% CO_2_, 37 °C. For 200-mL transfection in each 1-L flask, the plasmid DNA encoding C-terminal Twin-Strep-tagged recombinant human ODC was transfected into Expi293F^TM^ cells using FectoPRO^®^ transfection kit (Polyplus) according to manufacturer’s protocol. Then, enhancer was added immediately. After 24 h, the incubation temperature was lowered from 37 °C to 32 °C to slow cell growth rate while further enhancing protein expression yield^36^^,^^38^. Overexpression of recombinant human ODC1 was monitored for 24 to 48 h via IRES-driven GFP expression in Expi293^TM^ cells until transfection efficiency was reached to more than 60%. Lastly, the transfected cells were harvested and kept at −80 °C for long-term storage.

### Purification of Twin-Strep-tagged recombinant human ODC by Strep-Tactin chromatography

The entire procedure must be performed at 4 °C. Approximately 5 g of cell pellet was obtained from 200 mL of cell suspension in a 1-L flask. The cell pellet was resuspended in 10 volumes of running buffer, which contains 50 mM potassium pyrophosphate (K4P2O7), 500 mM NaCl, 5% glycerol and 0.1% Tween 20 at pH 7.5, along with protease inhibitor mix (1 µg/mL pepstatin A, 1 µg/mL aprotinin, 1 µg/mL leupeptin). In order to lyse cells, sonication was carried out by using a 500 W ultrasonic processor (Sonics & Materials Inc., VCX 500) for 60 cycles of 2 s “ON”/3 s “OFF” (2 min of sonication time) at 30% amplitude. Then, the cell lysates were centrifuged at 18,000 rpm for 30 min to remove the precipitate that might clog the chromatography column (BioRAD, 7372512). The column was filled with 4 mL of Strep-Tactin^®^ Sepharose^®^ resin (Iba, 2–1201-025) and equilibrated with 40 mL of the running buffer. Then, all the cell lysates were applied to the column by gravity flow. After the resin was settled, the column was washed with 50 mL of the running buffer once. Before collecting the eluate, Strep-Tactin resin was incubated in 10 mL of elution buffer, which is a mixture of the running buffer and 10 mM Desthiobiotin (Iba, 2–1000-005), for at least 15 min (no more than 30 min) to increase protein yields. Subsequently, specifically bound Twin-Strep-tagged human ODC homodimers were eluted with 10 mL of the elution buffer. Finally, the eluted fractions were concentrated with a 10 K MWCO centrifugal filter (Merk, UFC901024) and filtered with 0.45 µm Costar^®^ Spin-X^®^ centrifuge tube filter (Corning, CLS8162-96EA) before size-exclusion chromatography. The 3C-Avi-Twin-Strep tag at the C-terminus of the human ODC consists of a sequential Avi tag and Twin-Strep tag preceded by a human rhinovirus (HRV) 3C protease site^39^. The HRV 3C protease site allows removal of the purification tag as an option by digestion with Pierce^TM^ HRV 3C protease (Thermo Fisher Scientific, 88946) according to the manufacturer’s protocol. However, because small peptide tags usually do not affect protein properties^40^, we did not remove the purification tag which indeed did not greatly deteriorate the biological activity of the recombinant human ODC.

### Purification of recombinant human ODC homodimer by size-exclusion chromatography

The entire purification procedure was performed at 4 °C. Size-exclusion chromatography of recombinant human ODC homodimer was performed in a ÄKTA go chromatography system (Cytiva, 29383015) equipped with an ultraviolet (UV)-visible detector. Standard (BioRAD, 1511901) or sample solutions were injected into the HPLC system through a Rheodyne injector. The separation was achieved by using Superose^®^ 6 Increase 10/300 GL column (Cytiva, 29091596). The running buffer at a flow rate of 0.5 mL/min was used as a mobile phase to transfer and various proteins mixed in the eluate were discriminated according to their molecular weight. The chromatograms were monitored by UV detection at a wavelength of 280 nm. 0.5 mL of fractions were collected every minute for 1 h. Lastly, the fractions containing pure human ODC homodimers were combined. The purified protein concentration was measured by NanoDrop One spectrophotometer (Thermo Fisher Scientific) using absorbance at 280 nm. The purified recombinant human ODC was stored at −80 °C for long term storage.

### SDS-PAGE and Western blot analysis

For sodium dodecyl sulfate-polyacrylamide gel electrophoresis (SDS-PAGE) in discontinuous buffer systems, we utilised discontinuous SDS-PAGE gels consisting of a large-pore stacking gel (5% acrylamide) on top of a small-pore resolving gel (10% acrylamide). The protein fractions separated by 10% SDS-PAGE under reducing conditions were stained with Coomassie Blue (Abcam, ab119211). For western blot analysis, the protein fractions were separated by 10% SDS-PAGE under reducing conditions and then electrophoretically transferred onto polyvinylidene difluoride (PVDF) membranes using the iBlot^TM^ 2 Dry Blotting System (Thermo Fisher Scientific). After incubation with 5% skim milk in TBST (10 mM Tris, pH 8.0, 150 mM NaCl, 0.5% Tween 20) for 1 h at 25 °C, membranes were washed once with TBST and incubated with a 1:1000 dilution of rabbit monoclonal antibody against ODC (Abcam, ab97395) at 4 °C overnight. Then, membranes were washed with TBST five times and incubated with a 1:3000 dilution of horseradish peroxidase-conjugated goat anti-rabbit IgG (BioActs, RSA1221) for 1 h at 25 °C. Subsequently, blots were washed with TBST five times and developed with ECL substrate (BioRAD, 1705061). Chemiluminescent images were captured by ImageQuant LAS 500 chemiluminescence CCD camera (Cytiva).

### Human ODC enzyme activity assay

The ODC enzyme reaction was carried out thoroughly at 37 °C. Before measuring fluorescence (Excitation wavelength: 450 nm, Emission wavelength: 582 nm), all test samples and non-enzyme blanks were prepared at a final volume of 200 *µ*L/well in a 96-well plate with black flat bottom. The traditional ODC tandem assay and the optimised ODC tandem assay in a 96-well plate format are well described in Table 1. For the optimised ODC tandem assay in a 384-well plate format, 0.5 *µ*L of the reaction mixture was sampled and added to a well containing 49.5 *µ*L of CB6/DSMI solution. Assay buffer-dissolved 13.2 *µ*M CB6 and DMSO-dissolved 120 mM DSMI were diluted in the assay buffer to make 0.66 *µ*M CB6/4 *µ*M DSMI solution, thereby minimising DMSO sensitivity of the assay. The final assay solution in Figure 1(B) consisted of 25 *µ*L of enzyme buffer, 150 *µ*L of × *µ*M CB6/4 *µ*M DSMI solution and 25 *µ*L of 100 *µ*M ornithine dissolved in assay buffer, where × is varying concentrations at 0.5, 0.66, 0.75, 1, 1.25, 3.5 and 7. The final assay solution in Figure 1(C) consisted of 25 *µ*L of enzyme buffer, 150 *µ*L of × *µ*M CB6/4 *µ*M DSMI solution and 25 *µ*L of y *µ*M putrescine/z *µ*M ornithine dissolved in assay buffer, where y is varying concentrations at 0, 10, 20, 30, 40, 50, 60, 70, 80, 90 and 100 and *z* = 100 – *y*. The final assay solution in Figure 1(D) consisted of 1 *µ*L of DMSO (0, 0.1, 0.3, 1, 3, 10, 30 and 100%), 24 *µ*L of enzyme buffer, 150 *µ*L of 4 *µ*M DSMI solution and 25 *µ*L of 100 *µ*M ornithine dissolved in assay buffer. The final assay solution in Figure 1(E) consisted of 1 *µ*L of DMSO (0, 0.1, 0.3, 1, 3, 10, 30 and 100%), 24 *µ*L of enzyme buffer, 150 *µ*L of 0.66 *µ*M CB6/4 *µ*M DSMI solution and 25 *µ*L of 100 *µ*M ornithine dissolved in assay buffer. The fluorescence quenching caused by the production of putrescine, which was proportional to human ODC activity, was recorded with SpectraMax iD5 Multi-Mode Microplate Reader (Molecular devices). To correct for the background interferences, we calculated Δfluorescence by subtracting the averaged fluorescence value for non-ODC blanks from the fluorescence values for test samples with ODC. All experiments were performed in triplicate and data were presented as mean ± SEM (Standard Error of Mean). Data plotting and regression analysis were performed with GraphPad Prism software (Version 9.1.2, GraphPad Software).

**Table 1.** Traditional and optimised ODC tandem assay protocol in a 96-well microplate format.

Traditional assay		
For each well of a 96-well black plate (Enzyme reaction + Putrescine detection), add:		
1. ODC inhibition	25x DFMO dissolved in DW or DMSO	1 µL
	ODC in EB^a^ (178 ng/mL)	24 µL^c^
	Incubate at 37 °C for 15 min. Then, add:	
2. Stabilisation	0.66 µM CB6/4 µM DSMI in AB^b^	150 µL
	Incubate at 37 °C for more than 10 min in the dark, and add:	
3. ODC reaction (37 °C)	100 µM ornithine in AB	25 µL
4. Measuring fluorescence	Measure fluorescence (λ_ex_/λ_em_ = 450/582 nm) in a microplate reader at 3-min intervals for 1 hr during ODC reaction at 37 °C.	

Optimised assay		
For each well of a 96-well plate (Enzyme reaction), add:		
1. ODC inhibition	100x DFMO dissolved in DW or DMSO	1 µL
	ODC in EB (178 ng/mL)	99 µL^d^
	Incubate at 37 °C for 15 min. Then, add:	
2. ODC reaction (37 °C)	200 µM ornithine in AB	100 µL
3. Sampling	Sample 2 µL of reaction mixture at 5-min intervals for 1 hr during ODC reaction at 37 °C.	
For each well of a 96-well black plate (Putrescine detection), add:		
4. Stabilisation	The sampled reaction mixture	2 µL
	0.66 µM CB6/4 µM DSMI in AB	198 µL
	Incubate for more than 10 min in the dark.	
5. Measuring fluorescence	Measure fluorescence (λ_ex_/λ_em_ = 450/582 nm) in a microplate reader.	

^a^Enzyme buffer (EB): 50 mM Tris buffer containing 0.1 mM EDTA, 0.1 mM PLP, 2.5 mM DTT and 0.1% Tween 80 (pH 7.5).

^b^Assay buffer (AB): 50 mM Tris buffer (pH 7.5).

^c^For non-ODC blank, add 24 µL of EB instead.

^d^For non-ODC blank, add 99 µL of EB instead.

### Z-factor calculation

The Z-factor is defined in terms of four parameters: the means (µ) and standard deviations (*σ*) of both the positive (*p*) and negative (*n*) controls (µ_p_, µ_n_ and *σ*_p_, *σ*_n_). Given these values, the Z-factor is defined as:

$$\text{z}-\text{factor}=\text{1}-\text{3}\left(\sigma_{\text{p}}+\sigma_{\text{n}}\right)/|\mu_{\text{p}}-\mu_{\text{n}}|$$

## Results and discussion

### CB6/DSMI complex does not tolerate the presence of high concentrations of DMSO

To optimise the tandem assay for HTS of ODC inhibitors, we firstly obtained an optimal stock concentration of CB6 for a fixed stock concentration (4 µM) of DSMI by fluorescence imaging assay. We observed that fluorescence kept increasing upon increasing concentration of CB6 (0.5–7 µM) (Figure 1B). Then, we performed a standard calibration with putrescine at varying concentrations (0–100 µM) with each CB6 concentration and obtained an ideal inverse linear relationship between fluorescence and putrescine concentration with CB6 at 0.66 µM (Figure 1C). These results indicate that 0.66 µM CB6 is optimal for DSMI at 4 µM to detect putrescine production from ornithine.

Next, to test the possibility of DMSO-dependent disruption of CB6/DSMI complex, we firstly monitored time-dependent fluorescence changes of DSMI with DMSO at different concentrations (0–0.5%) (Figure 1D). It was found that DSMI itself did not show any DMSO-dependent fluorescence quenching. In contrast, CB6/DSMI complex showed a significant fluorescence quenching in the presence of DMSO at high concentrations (0.015–0.5%) (Figure 1E). In particular, the fluorescence quenching of CB6/DSMI complex almost disappeared in the presence of DMSO at low concentrations (0–0.005%). Based on the DMSO concentration versus relative fluorescence curve measured at 30-min time point (Figure 1E), we obtained a half-maximal inhibitory concentration (IC_50_) of 0.056% DMSO (Figure 1F) or 7.832 mM DMSO (Figure 1G). These results are consistent with previous work reporting a binding constant of DMSO to CB6 (K_a_ = 1.2 × 10^3^ M^–1^)^30^, implicating that DMSO can be another competitor of DSMI for binding to CB6. Furthermore, these results open up the possibility of improving the ODC tandem assay by decreasing DMSO concentration below 0.005%, at which the fluorescence quenching of CB6/DSMI complexes becomes insignificant.

### Mass production of recombinant human ODC by using mammalian cell-line

**Figure 2.**
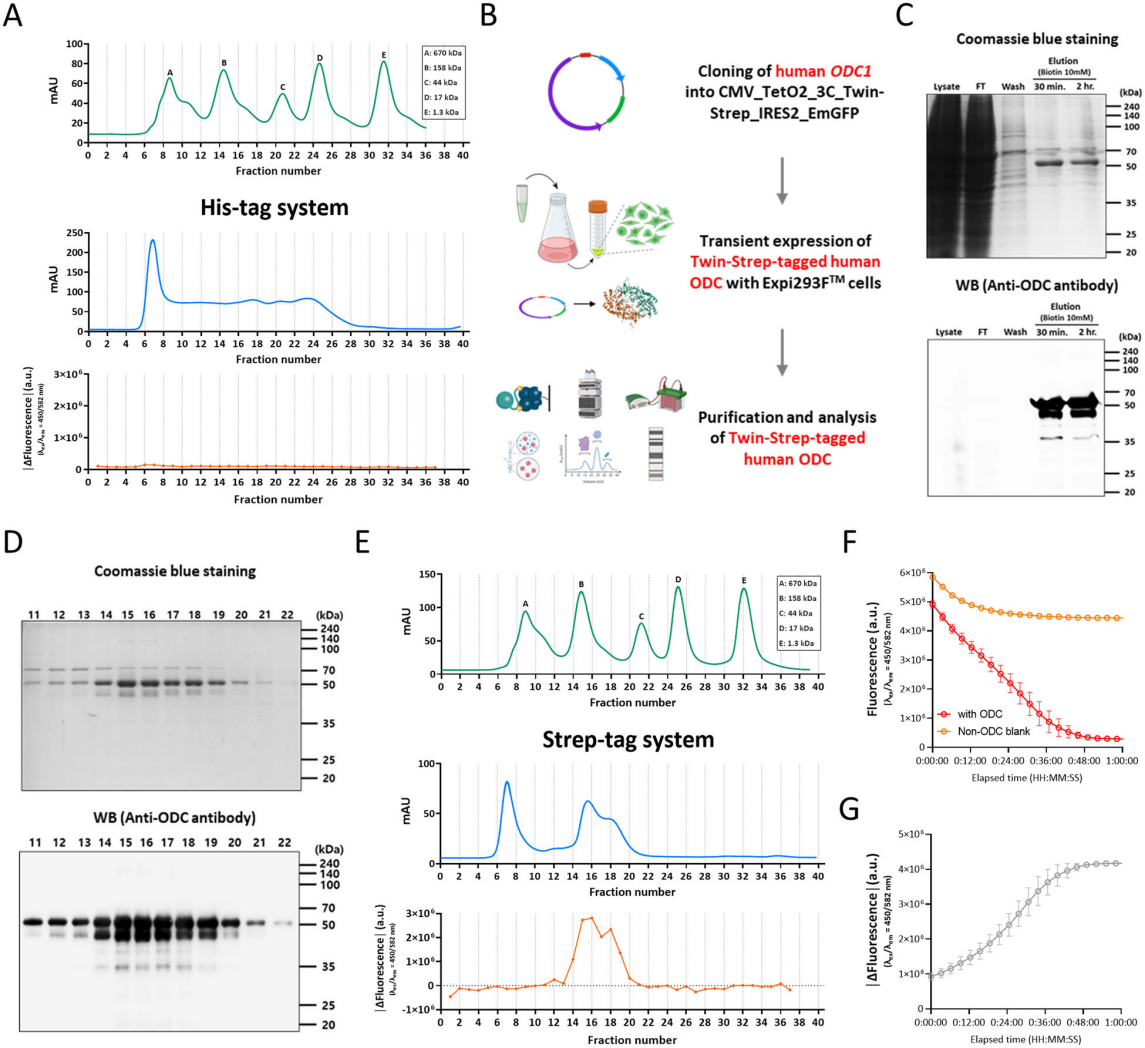
Human cell-line-based mass production of recombinant human ODC homodimers. A. Chromatograms of standard solution (top) and sample solution purified via His-tag system (middle) and ODC enzyme activity of each fraction from size-exclusion chromatography (bottom). B. Workflow for Expi293F^TM^-based overexpression of Twin-Strep-tagged human ODC followed by Strep-Tacin chromatography and size-exclusion chromatography. C. Coomasie blue staining (top) and western blot analysis (bottom) of ODC-immunoreactive protein eluates from Strep-Tactin chromatography. D. Coomasie blue staining (top) and western blot analysis (bottom) of ODC-immunoreactive protein fractions (11th to 22nd) from size-exclusion chromatography. E. Chromatograms of standard solution (top) and sample solution purified via Strep-tag system (middle) and ODC enzyme activity of each fraction from size-exclusion chromatography (bottom). F. Time-course plots of the fluorescence of CB6/DSMI with ODC enzyme reaction (in red) and without ODC enzyme reaction (in orange, non-ODC blank). G. A time-course plot of ODC enzyme reaction corrected for background and positively sloped.

To produce recombinant human ODC with appropriate folding and PTMs in large quantities for HTS, we attempted to utilise a protocol for overexpression of C-terminal 6xHis-tagged human ODC in Expi293F^TM^ cells, followed by Ni-NTA affinity chromatography and size-exclusion chromatography as previously described^36^. Unexpectedly, we were not able to obtain any fraction containing biologically active human ODC (Figure 2A, bottom panel).

To overcome this problem, we used Strep-tag system, which is known to be compatible with any expression system including mammalian cells^41^. We performed overexpression of Twin-Strep-tagged human ODC in Expi293F^TM^ cells followed by a two-step protein purification (Figure 2B). Firstly, the Twin-Strep-tagged human ODC was purified from cell lysate via Strep-Tactin affinity chromatography. The elution of Twin-Strep-tagged human ODC was carried out with elution buffer containing 10 mM biotin. The eluted recombinant human ODC exhibited a size of 51 kDa on SDS-PAGE gel under reducing conditions (Figure 2C). Secondly, we further purified the eluted fractions by size-exclusion chromatography (Figure 2D), because ODC is catalytically active only in a homodimeric form^23^^,^^32^^,^^42^. Considering the molecular weight of human ODC homodimer (102 kDa), we confirmed that sample peak fractions (14th to 20th fractions) between standard peak B (158 kDa) and C (44 kDa) contained biologically active human ODC homodimers (Figure 2E, top and middle panels) by performing the ODC tandem assay (Figure 2E, bottom panel), Coomassie Blue staining and western blot analysis (Figure 2D). These results indicate that we successfully obtained biologically active human ODC homodimers for HTS.

Finally, the sample peak fractions were combined and concentrated. To evaluate the activity of the combined and concentrated human ODC, the tandem assay was performed for measuring the time-course of human ODC reaction (Figure 2F). To plot a positively sloped time-course of ODC enzyme reaction, we calculated the absolute values of Δfluorescence (|Δfluorescence|). The resulting ODC reaction plot (Figure 2G) showed that ODC enzyme reaction was saturated at 40 min, which limited the dynamic range of the assay beyond 40 min. In summary, we have successfully established the protocol for high-yield synthesis and purification of the recombinant human ODC homodimers by using human cell-line, yielding 0.7 mg of recombinant human ODC from 5 g of cell pellet for one batch. In addition, we found that Strep-tag system, not His-tag system, was suitable for purification of recombinant human ODC produced by mammalian expression system.

### Optimisation of ODC tandem assay by separating enzyme reaction and putrescine detection

**Figure 3.**
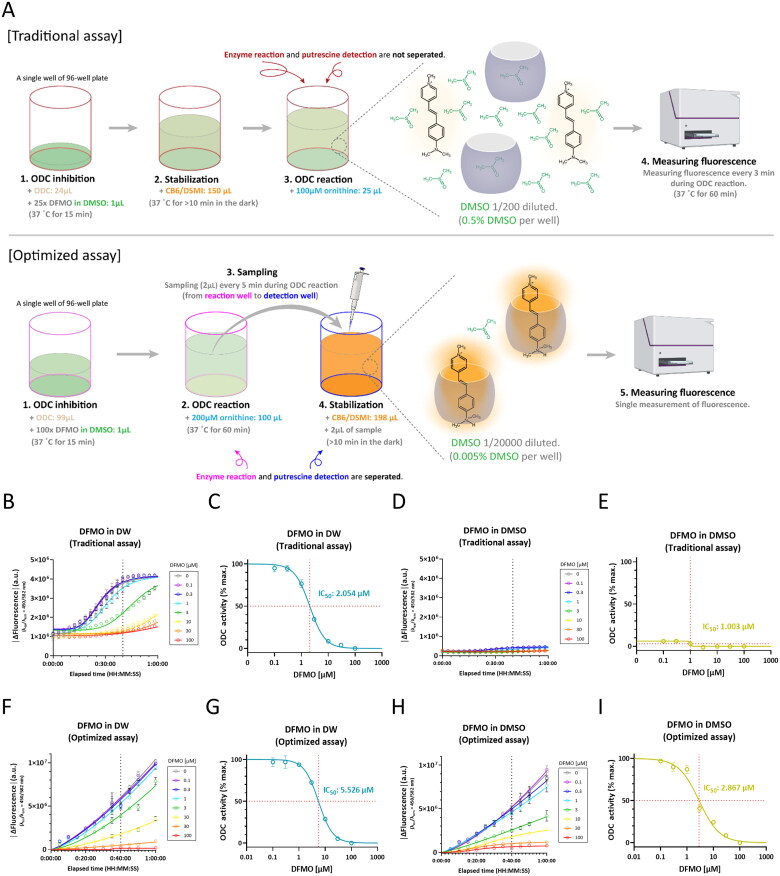
Optimised ODC tandem assay minimises the DMSO-dependent fluorescence quenching of CB6/DSMI and further expands the dynamic range. A. Workflows for traditional ODC tandem assay (top) and optimised ODC tandem assay (bottom). B. Time-course plots of ODC enzyme reaction with DFMO dissolved in DW at varying concentrations (traditional assay). C. A dose-response curve of human ODC inhibition by DFMO dissolved in DW (traditional assay). D. Time-course plots of ODC enzyme reaction with DFMO dissolved in DMSO at varying concentrations (traditional assay). E. A dose-response curve of human ODC inhibition by DFMO dissolved in DMSO (traditional assay). F. Time-course plots of ODC enzyme reaction with DFMO dissolved in DW at varying concentrations (optimised assay). G. A dose-response curve of human ODC inhibition by DFMO dissolved in DW (optimised assay). H. Time-course plots of ODC enzyme reaction with DFMO dissolved in DMSO at varying concentrations (optimised assay). I. A dose-response curve of human ODC inhibition by DFMO dissolved in DMSO (optimised assay).

Using the purified human ODC, we attempted to improve the traditional ODC tandem assay by circumventing the DMSO sensitivity of CB6/DSMI complex. The traditional ODC tandem assay in a 96-well microplate format allows real-time monitoring of ODC activity by fluorescence spectroscopy^27^. For the real-time monitoring, ODC reaction and putrescine detection have to occur simultaneously, such that ODC, CB6/DSMI complex and ornithine were sequentially added in the same well of the 96-well black microplate (Table 1, top panel). In the beginning of the traditional assay, 1 *µ*L of 25x DFMO stock dissolved in DMSO and 24 *µ*L of ODC solution were added. After adding 150 *µ*L of CB6/DSMI and 25 *µ*L of 100 *µ*M ornithine solution, DMSO was diluted to a final concentration of 0.5% at which DMSO was highly likely to displace DSMI from CB6 and result in a concomitant decrease in fluorescence intensity even before putrescine was formed (Figure 3A, top panel). To investigate whether DMSO dissolving DFMO distorts the traditional ODC tandem assay, we recorded the time-courses of human ODC reactions with DFMO dissolved in distilled water (DW) or DMSO at different concentrations (Figure 3B and D). It was found that 0.5% DMSO resulted in a 15-fold reduction in dynamic range (Figure 3D). For dose-response curves of DFMO dissolved in DW or DMSO, all the values were normalised with respect to the value obtained with 0 *µ*M DFMO in DW (Figure 3C and E). IC_50_ of DFMO in DW was 2.054 *µ*M and Hill slope was −1.54 in the traditional assay (Figure 3C). IC_50_ of DFMO in DMSO was 1.003 *µ*M in the traditional assay. However, we were not able to calculate Hill slope of the dose-response curve of DFMO in DMSO, because a distorted dose-response curve was obtained due to the limited dynamic range (Figure 3E). These results indicate that the traditional ODC tandem assay highly distorts the dose-response curve due to DMSO-dependent quenching of CB6/DSMI complex.

To overcome this limitation, we separated ODC reaction and putrescine detection to develop an optimised DMSO-tolerant assay (Table 1, bottom panel). In the beginning of the optimised assay, 1 *µ*L of 100x DFMO stock dissolved in DMSO and 99 *µ*L of ODC solution were added. After adding 100 *µ*L of 200 *µ*M ornithine solution, 2 µL of the reaction mixture was sampled to further dilute DMSO by distinguishing putrescine detection from enzyme reaction. 2 µL of the sampled reaction mixture was transferred into a new well of 96-well black plate filled with 198 µL of CB6/DSMI solution. DMSO was diluted to a final concentration of 0.005% at which DMSO-dependent CB6/DSMI quenching is negligible (Figure 3A, bottom panel). To ensure enough production of putrescine in 2 µL of the sampled reaction mixture, we increased the quantity of ODC and ornithine in the reaction mixture (Figure 3A). To investigate whether the optimised ODC tandem assay tolerates DMSO dissolving DFMO, we recorded the time-courses of human ODC reactions with DFMO dissolved in DW or DMSO at different concentrations (Figure 3F and H). Dose-response curves obtained by the optimised assay were plotted as we did in the traditional assay (Figure 3G and I). IC_50_ of DFMO in DW was 5.526 *µ*M and Hill slope was −1.586 in the optimised assay (Figure 3G). Unlike the traditional ODC tandem assay, we could obtain IC_50_ of DFMO in DMSO (2.867 *µ*M) and Hill slope (-1.122) of the dose-response curve by the optimised assay (Figure 3I). Surprisingly, the optimised assay showed a more than 2-fold increase in the dynamic range compared to the traditional assay (Figure 3B, F and H), which allows better detection of subtle but significant signals. Taken together, the optimised ODC tandem assay tolerates DMSO and further expands the dynamic range.

### Validation of traditional ODC tandem assay and optimised ODC tandem assay by calculating Z-factor

**Figure 4.**
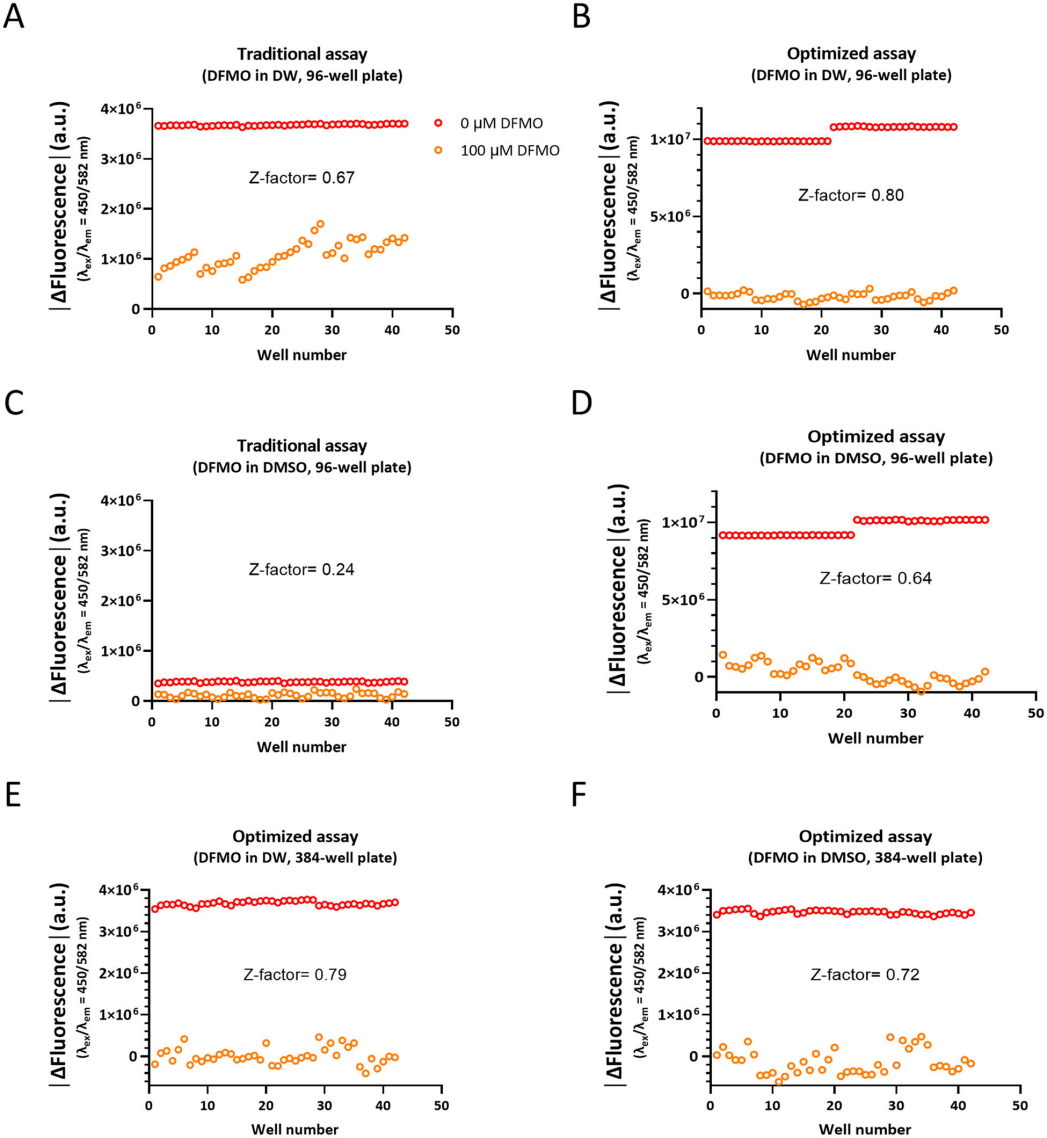
Validation of traditional ODC tandem assay and optimised ODC tandem assay by calculating Z-factor. (A–D) The scatter plots showing the positive control (100 µM DFMO, ornage circle) and negative control (0 µM DFMO, red circle) data for the Z-factor calculation in a 96-well microplate format. 42 samples were used for calculating the means and standard deviations. (E–F) The scatter plots showing the positive control (100 µM DFMO, ornage circle) and negative control (0 µM DFMO, red circle) data for the Z-factor calculation in a 384-well microplate format. 42 samples were used for calculating the means and standard deviations.

To evaluate the quality of the HTS assays, Z-factor was calculated according to the method described by Zhang et al., 1999^43^. The Z-factor with value greater than 0.5 has been considered as an indicative of an excellent HTS assay. The Z-factor in our study was defined as the degree of separation between the mean value for the positive control (100 µM DFMO) and the mean value for the negative control (0 µM DFMO). In a 96-well plate format, the Z-factor for the traditional assay with DFMO in DW was 0.67 (Figure 4A), which was comparable to 0.8 for the optimised assay (Figure 4A and B). In contrast, the Z-factor for the traditional assay with DFMO in DMSO was 0.24 (Figure 4C), which could be considered as a marginal assay, whereas the optimised assay showed a significantly improved Z-factor of 0.64 (Figure 4D). These results indicate that the optimised ODC tandem assay with DMSO solvent can be an excellent assay for HTS. Finally, we demonstrated that our optimised ODC tandem assay can be applied to HTS in a 384-well plate format. The Z-factors for the optimised assay with both DFMO in DW and DFMO in DMSO were above 0.5 (Figure 4E and F). Taken together, our optimised ODC tandem assay has been validated for HTS not only in 96-well, but also in 384-well plate format.

### The dynamic range of ODC tandem assay is enhanced by the use of 10 mM Tris buffer over 50 mM Tris buffer

**Figure 5.**
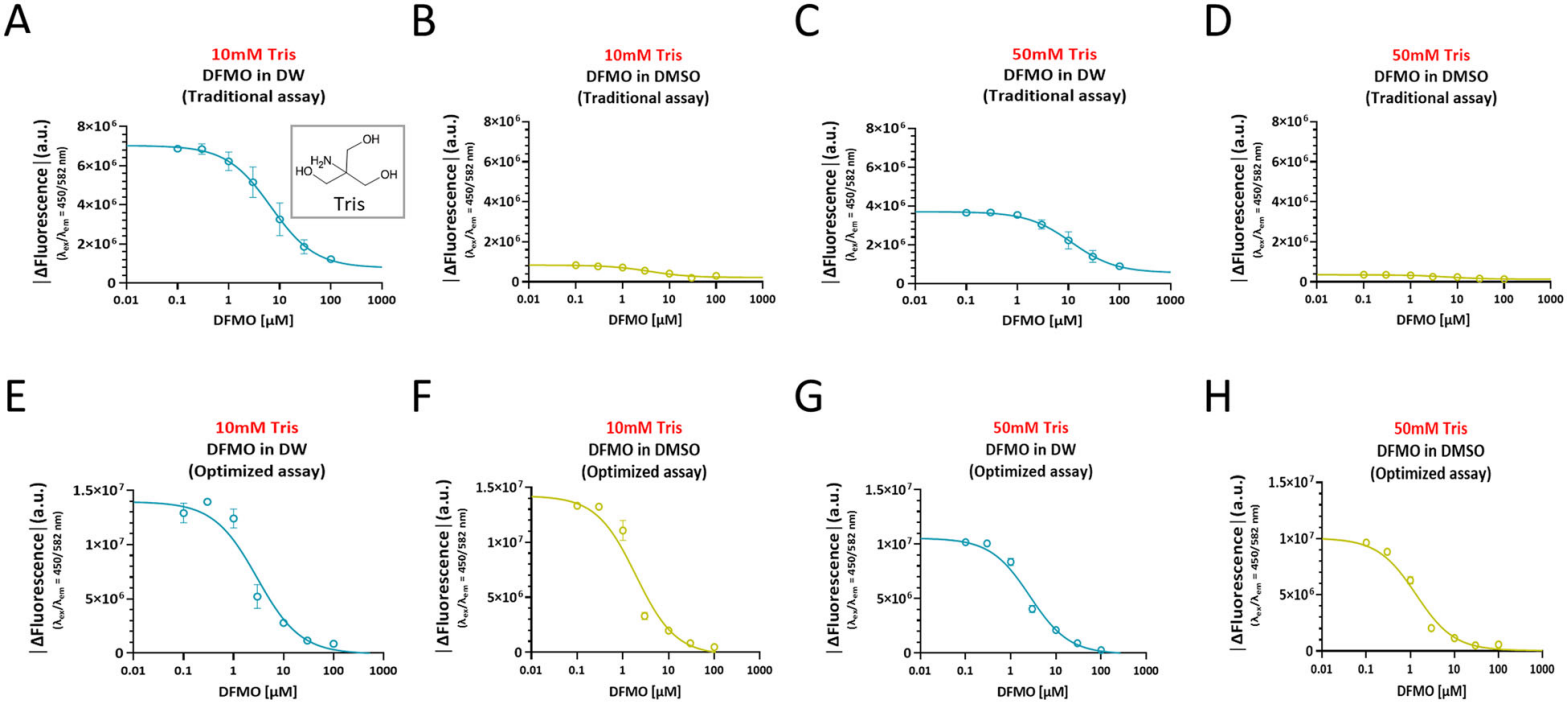
The dynamic range of ODC tandem assay is enhanced by the use of 10 mM Tris buffer over 50 mM Tris buffer. Dose-response curves of human ODC inhibition by DFMO dissolved in DW or DMSO. (A–B) Traditional assay with 10 mM Tris buffer. (C–D) Traditional assay with 50 mM Tris buffer. (E–F) Optimised assay with 10 mM Tris buffer. (G–H) Optimised assay with 50 mM Tris buffer.

In this study, we used a higher concentration of Tris buffer (50 mM), compared to 10 mM Tris buffer used by Nilam et al.,^27^. To test the possibility that Tris is competing with other components for binding to CB6, we directly compared 10 mM Tris buffer and 50 mM Tris buffer in the traditional and the optimised ODC tandem assays. We found that the traditional assay showed DMSO-dependent fluorescence quenching regardless of Tris concentration (Figure 5A–D). In marked contrast, the optimised assay showed no DMSO sensitivity in both 10 mM and 50 mM Tris buffers (Figure 5E–H). Although DMSO sensitivity of the traditional assay was independent of Tris concentration, we found that 10 mM Tris buffer showed 1.5 to 2-fold larger dynamic range than 50 mM Tris buffer in both the traditional and the optimised assays (Figure 5A, C, E and G). These results indicate that Tris is likely to induce a mild fluorescence quenching, which should be taken into account in future optimizations of the assay.

## Conclusions

We have successfully upgraded the ODC tandem assay which was originally developed for HTS but has not been used frequently for HTS due to its DMSO intolerance. Our DMSO-tolerant ODC tandem assay can be adapted to other tandem enzyme assays^44^ for the purpose of screening DMSO-dissolved compound libraries. Furthermore, our human cell-line-based protein production system ensures a stable supply of active recombinant human ODC homodimer in large quantities for HTS. In conclusion, our DMSO-tolerant ODC tandem assay should prove to be useful in developing more effective ODC modulators for the treatment of human diseases, such as cancer and AD.
